# Principles of dose finding studies in cancer: a comparison of trial designs

**DOI:** 10.1007/s00280-012-2059-8

**Published:** 2013-01-09

**Authors:** Thomas Jaki, Sally Clive, Christopher J. Weir

**Affiliations:** 1 Medical and Pharmaceutical Statistics Research Unit, Lancaster University, Lancaster, UK; 2 Edinburgh Cancer Centre, Western General Hospital, Edinburgh, UK; 3 Medical Research Council Hub for Trials Methodology Research, Centre for Population Health Sciences, University of Edinburgh Medical School, Teviot Place, Edinburgh, EH8 9AG UK

**Keywords:** 3 + 3 design, Bayesian method, Clinical trial, Phase I, Continual reassessment method, CRM, Curve free

## Abstract

**Purpose:**

One key aim of Phase I cancer studies is to identify the dose of a treatment to be further evaluated in Phase II. We describe, in non-statistical language, three classes of dose-escalation trial design and compare their properties.

**Methods:**

We review three classes of dose-escalation design suitable for Phase I cancer trials: algorithmic approaches (including the popular 3 + 3 design), Bayesian model-based designs and Bayesian curve-free methods. We describe an example from each class and summarize the advantages and disadvantages of the design classes.

**Results:**

The main benefit of algorithmic approaches is the simplicity with which they may be communicated: it may be for this reason alone that they are still employed in the vast majority of Phase I trials. Model-based and curve-free Bayesian approaches are preferable to algorithmic methods due to their superior ability to identify the dose with the desired toxicity rate and their allocation of a greater proportion of patients to doses at, or close to, that dose.

**Conclusions:**

For statistical and practical reasons, algorithmic methods cannot be recommended. The choice between a Bayesian model-based or curve-free approach depends on the previous information available about the compound under investigation. If this provides assurance about a particular model form, the model-based approach would be appropriate; if not, the curve-free method would be preferable.

## Introduction

Ethical considerations [1] require the use of efficient trial designs in order to optimize the balance of risk versus benefit for participants. In Phase I cancer studies, this would include minimizing the numbers of patients allocated to ineffective or excessively toxic doses, while addressing the principal aim of identifying the best dose of a treatment to recommend for further evaluation in a Phase II trial. In this article, we focus on the identification of the maximum tolerated dose (MTD), which remains a key factor in this decision. In practice, additional aspects such as the biological level of anti-cancer activity of a dose would also be considered. The 3 + 3 trial design [2, 3], which has been widely implemented, has substantial limitations: we describe two alternative classes of dose-escalation strategy, using specific examples [4–6] drawn from the many designs available to illustrate their superiority to the 3 + 3 design on several relevant study design criteria.

Phase I studies play an extremely important role in the development of a cancer treatment as the agent is often given to humans for the first time. As a result, conservative approaches that tend to start at doses much lower than the anticipated highest safe dose and slowly approach the dose of interest from below are usually employed. The population studied is not necessarily the population to be treated and the questions are many despite the subjects being few. Consequently much uncertainty about dose, safety and efficacy will remain afterward. These studies are, however, critical to successful drug development as the decision on whether to continue and the design of any subsequent trials depend on the outcome of this first study.

Phase I dose-escalation studies in cancer traditionally enroll late stage patients for whom other therapies have failed. Due to the narrow therapeutic index of cytotoxic drugs and the variable toxicities that may occur at the therapeutic dose of newer targeted anti-cancer drugs, the MTD has been considered a reasonable basis on which to determine an appropriate dose for further clinical use. Hence, in addition to investigating the pharmacokinetics, toxicities and biological activity of a drug, Phase I cancer studies aim to find the MTD, the highest dose that can safely be administered. More precisely, one seeks the dose that has an acceptable risk of dose-limiting toxicity (DLT). In this context, a DLT is a serious adverse event that impairs usual activities and requires therapeutic intervention [7–9]. Late toxicities or lower graded, cumulative or additive toxicities that do not individually meet the DLT criteria also contain information on the safety and activity of a drug; however, the designs used to date do not take account of such events [10]. Advances in this area would clearly have potential to enhance decision making at the end of Phase I.

Commonly the dose at which the probability of a DLT is π (for 0 < π < 1) is called the TD100π. For example, the TD25 would refer to the dose associated with a 25 % toxicity risk. The rationale for seeking the TD100π is to ensure a safe dose is identified for further study and is based on the common assumption, specifically for cytotoxic compounds, that efficacy increases with toxicity. The latter implies that finding the highest tolerable dose will ensure that the most efficacious dose is investigated subsequently. First, cycle toxicity is a critical and immediate measure that can guide the decision on whether the dose may be escalated safely, and the use of this rather than later efficacy outcomes ensures minimal trial duration.

**Fig. 1:**
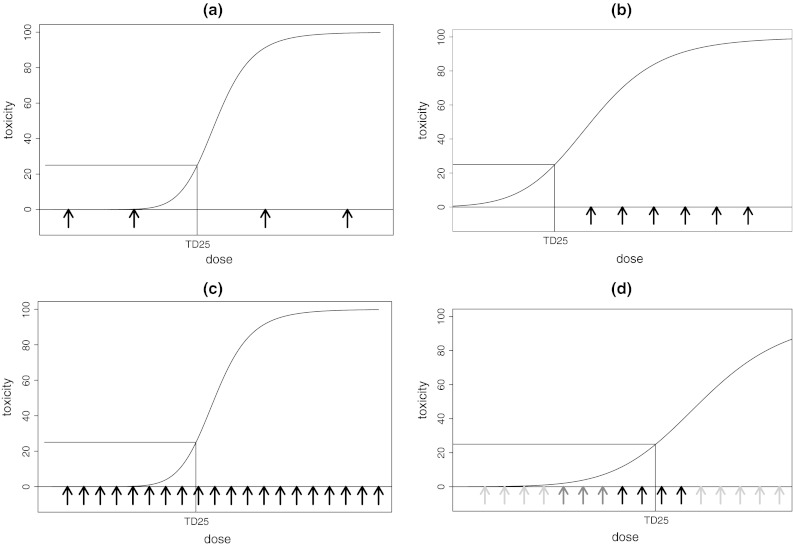
Schematic illustration of different problems in finding the TD25, the dose at which the underlying risk of dose-limiting toxicity is 25 %. **a** Four doses, none of which matches the target toxicity rate; (**b**) six doses, all lying above the desired toxicity level; (**c**) many doses, allowing accurate estimation of the TD25; (**d**) many doses, with increased efficiency through less frequent use of excessively toxic or ineffective doses

A key ingredient of any dose-escalation study is the set of doses to be explored. Figure 1 illustrates four different dose schedules for a given, hypothetical, dose–toxicity relationship. Each graph depicts dose versus toxicity risk and assumes an increasing relationship between them. The arrows indicate the doses available and it is assumed that a toxicity risk of 25 % is acceptable. Figure 1a has very low risk of toxicity for the lower two doses while the upper two doses have a toxicity risk close to 100 %. Such a dose schedule therefore only allows the conclusion that the TD25 lies somewhere between the middle two doses; neither of the doses available is, however, close to the desired toxicity risk. In Fig. 1b, all the doses lie above the desired toxicity level. In this situation, not only does none of the doses have an acceptable toxicity risk, but we cannot even be sure that any dose of the compound is safe. Similarly, if all doses were below the desired TD25, we would not know if any dose corresponded to the TD25 and hence whether a biologically active dose had been attained.

Figure 1c shows the advantage of allowing many different doses. It is clear that such a dose schedule allows much more accurate TD25 estimation as the interval between doses is much smaller. While adding doses to allow for more accurate estimation is appealing, doing so requires a different dose-escalation strategy to the commonly employed methods in order to limit the sample size and avoid many patients receiving doses well below those expected to have either toxicity or efficacy. Figure 1d represents an ideal situation where many doses are available, but excessively toxic or ineffective doses represented by lighter shaded arrows are explored less often or possibly even skipped altogether.

Traditionally, dose escalation starts at a low dose defined preclinically and slowly escalates dose in decreasing increments according to a predetermined strategy (the modified Fibonacci sequence, in which the dose increments for succeeding dose levels are 100, 67, 50, 40 % then 33 % for all subsequent levels [11]). Alternative escalation strategies [12] include accelerated titration designs [3], pharmacokinetically guided dose escalation [13] and continual reassessment or Bayesian methods [4, 14–16] which have been successfully incorporated into Phase I trial design [17, 18].

From here on, we assume that a sensible selection of doses is available but stress that inclusion of more potential doses within the range of interest may allow more accurate estimation of the highest tolerable dose. To avoid “wasting” patients at doses that are no longer of interest based on the data gathered so far in the trial, skipping doses [6] or the use of single patients at early dose levels [3] could be considered.

There exists a vast literature on dose-escalation methods for Phase I trials in cancer, yet very few of them have been used in practice. By far the most popular approach to dose escalation is the 3 + 3 design [2] which, according to Le Tourneau et al. [12], was used in 96.7 % of Phase I trials published in 2007 and 2008. A similar finding was made by Rogatko et al. [19] when they investigated the use of Bayesian designs in Phase I cancer trials. Only 20 (1.6 %) of 1,235 trials followed a Bayesian design despite the existence of around 100 publications demonstrating the statistical properties of such designs.

There are three general approaches to dose finding studies in cancer. We will introduce their main features and compare their advantages and disadvantages. In particular, we will show that the most popular design in practice has severe shortcomings when it comes to identifying the dose of interest.

## Design alternatives

### Algorithmic approaches

**Fig. 2:**
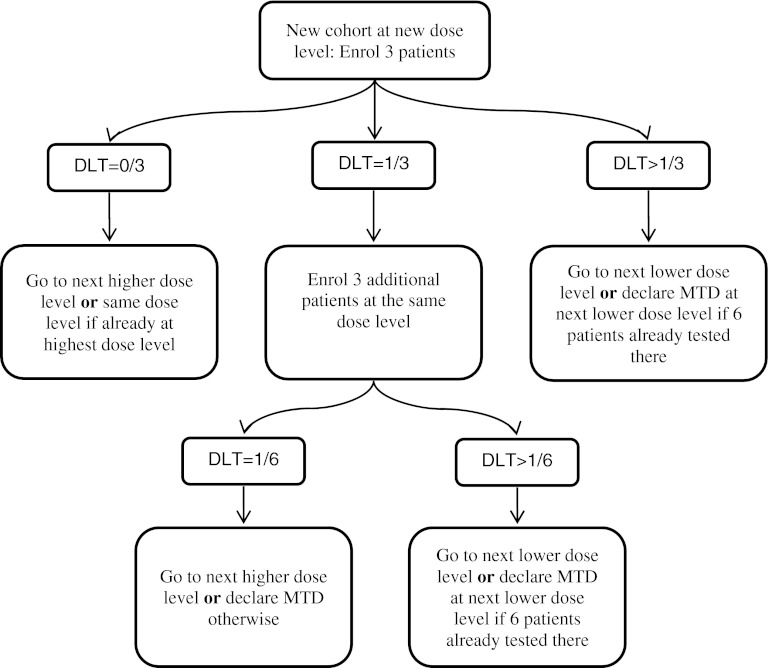
Schematic display of one version of the 3 + 3 design

Rule-based dose-escalation methods include the Storer up-and-down design [3] and the frequently applied 3 + 3 design (Fig. 2). Although several variants of this long-established approach have been published, the earliest source is a book chapter arising from the proceedings of a clinical pharmacology course held in Brussels in May 1972 [2]. The main principle of algorithmic designs is that a small group of patients is treated at a given dose and, dependent on the observed number of toxicities, a decision is made on to whether to study a further group of patients at the next dose up the scale, to study more patients at the same dose or to stop the trial.

The 3 + 3 design, for example, enters three patients into the trial one at a time at intervals of seven days or more and treats them at the chosen starting dose. The core features of this design, as outlined by Storer [3], are:

- If no dose-limiting toxicity (DLT) occurs in the first cycle of any of the initial group of 3 patients treated at a dose, the dose should be escalated for the next group of patients.
- If two or more of the 3 patients treated at a given dose experience a DLT, the trial should stop.
- If one patient of the 3 treated at a given dose level experiences a DLT, a further 3 patients are treated at the same dose level. If a DLT has occurred in exactly one of these 6 patients, escalation may continue as in (a); otherwise, the trial should stop.

A frequent variation allows the dose to be reduced if more than one DLT is observed within the first three patients studied at a particular dose.

Following completion of the trial according to this design, the MTD is defined as either the actual dose at which the trial was stopped or the next lower dose, possibly depending on the frequency and severity of toxicity observed in the patients evaluated in the final group [3]. A number of adaptations of this design attempt to reduce the number of patients treated at the lowest dose levels [20]. Such accelerated titration designs [18, 21] include single-patient dose levels and more rapid dose escalation (e.g., dose doubling) until the first DLT is observed, after which the dose-escalation scheme would then revert to the modified Fibonacci sequence.

### Model-based Bayesian methods

Bayesian modeling combines prior knowledge of the drug with the observed data from the current trial to provide updated information about the distribution of the trial outcome of interest. Bayesian methods have been incorporated into a number of early phase clinical trial designs, the best known being the continual reassessment method (CRM) [4, 5]. In contrast to the algorithmic approach of the 3 + 3 design, the continual reassessment method aims to identify the dose at which the proportion of patients experiencing a DLT reaches a specific target level (e.g., 25 %). Furthermore, the investigator performs repeated analyses on all of the data gathered to date—hence the term continual reassessment—rather than simply observing the data recorded at the current dose, as is the case for the 3 + 3 design.

The CRM uses a one-parameter Bayesian model which assumes that the probability of toxic response increases with dose: as we move from one dose up to the next higher dose, the toxicity risk at the higher dose is greater than or equal to that at the previous dose. Before the trial has commenced, Bayesian modeling requires a prior distribution for the dose–toxicity curve parameters to be specified [22], representing the expected shape of the dose–toxicity relationship. In the original version of the CRM, a simple one-parameter prior distribution is recommended; subsequent developments allow the prior to be informed by data (such as information from pre-clinical studies) or expert opinion (see for example [23] for alternatives). The model may be updated as soon as new data become available on previously included patients. Following this the next patient, or group of patients, is treated at the dose, based on the evidence to date, that has an estimated probability of toxicity closest to the target level.

Once all patients have been treated and followed up, the MTD is taken to be the dose at which the estimated toxicity probability is closest to the target level. Although the originally proposed CRM method allows for the initial dose to be above the lowest available dose level, in practice, a modified version that enforces dose escalation to start from the lowest dose [24] is usually employed. Stopping rules have been developed for the CRM [25] to allow early discontinuation of the trial in the situation where all doses are found to be excessively toxic.

Alternative Bayesian model-based approaches include escalation with overdose control (EWOC) designs [16] and the TITE-CRM extension of the CRM which allows the trial to be completed more quickly by incorporating time to toxicity event data [26].

### Curve-free Bayesian approaches

This class of escalation strategies includes work by Gasparini and Eisele [27] and Whitehead et al. [6] and aims to minimize the number of assumptions within the dose–toxicity modeling. No assumption is made about the form of the relationship between dose and toxicity except that, as in the CRM, the probability of toxic response increases with dose. The risk of toxicity is modeled directly, resulting in an easy to interpret table of probabilities for each risk level.

The possible levels of toxicity risk at a dose are described qualitatively as very safe, safe, ideal, risky or toxic. The numerical probability value that corresponds to each of these descriptors depends on the target toxicity level. The “ideal” risk category has exactly the target toxicity probability, while the “safe” and “very safe” categories have progressively lower risks of toxicity and the “risky” and “toxic” categories have progressively higher risks. For example, in a study aiming to identify the dose which has toxicity rate 25 %, the descriptors very safe, safe, ideal, risky and toxic might be assigned the probability values 0.05, 0.15, 0.25, 0.4 and 0.65, respectively.

The prior distribution summarizing previous knowledge of the expected risk at each dose level, required under the Bayesian framework, is informed by investigator opinion. As data accumulate during the trial, the updated probabilities of each risk level being associated with each dose are calculated. The dose to be allocated to the next patient enrolled in the trial is selected to avoid doses that have anything other than a small chance of being “toxic” and to target the dose that has the greatest chance of being “ideal”. Table 1 illustrates the probabilities of each level of toxicity during a hypothetical trial. Here, 25 % is the target toxicity rate, and dose level 7 is the one with the highest probability of having that toxicity rate.

**Table 1:** Example of risk level probabilities generated by the Bayesian curve-free approach

Dose level	Risk of toxicity				
	5 % “very safe”	15 % “safe”	25 % “ideal”	40 % “risky”	65 % “toxic”
1	0.48	0.44	0.08	0.00	0.00
2	0.40	0.48	0.12	0.00	0.00
3	0.33	0.52	0.15	0.00	0.00
4	0.25	0.55	0.19	0.01	0.00
5	0.17	0.54	0.28	0.01	0.00
6	0.08	0.49	0.40	0.03	0.00
7	0.01	0.28	**0.61**	0.10	0.00
8	0.01	0.14	0.40	0.33	0.12
9	0.01	0.05	0.19	0.40	0.35

Bold value indicates the dose level with the highest probability of having the “ideal” toxicity rate

At the end of the trial, one of three approaches may be used to determine which dose to take forward for further study. The first method bases the decision on the final table of updated risk level probabilities. If the dose with a toxicity risk of 25 % was being sought, then the dose with the highest probability of having a toxicity risk of 25 % would be recommended. In the second, perhaps more realistic, strategy, the table of updated risk level probabilities would form just part of the information being considered by investigators when deciding what dose to recommend. The complete study data set will contain far more information than DLT occurrences: pharmacokinetic and clinical data and the expert opinion of investigators could also inform the recommendation. The third approach would be to take the data set and apply any appropriate method of statistical analysis, independently of the dose allocation method used in the trial.

## Discussion

The merits and shortcomings of the three classes of dose-escalation procedures introduced above will now be discussed. A broad range of important criteria, including statistical and practical aspects, to consider when evaluating a dose-escalation procedure will be considered under five headings. The weighting for each of the criteria will depend on the specific trial and consequently it is unlikely that each point will carry equal weight across all trials. We have summarized this in Table 2 where we have graded the performance of the dose-escalation procedure for each item under the five headings qualitatively as poor, intermediate, good or not applicable.

**Table 2:** Characteristics of dose-escalation strategies

Issues to consider	Algorithmic	Model based	Curve free
1. Statistical properties			
1a Does method provide estimate of a relevant parameter and allow the precision of the estimate to be quantified?	Poor	Good	Good
1b Does precision increase with sample size?	Poor	Good	Good
1c Reliably arrives at correct decision on dose with target toxicity risk	Poor	Good	Good
2. Simplicity			
2a Non-technical explanation of method	Good	Poor	Intermediate
2b Statistical complexity	Not applicable	Intermediate	Poor
3. Intuitive dose recommendations	Good	Intermediate	Good
4. Flexibility			
4a Target toxicity rate	Poor	Good	Good
4b Accommodating underlying shape of dose–response	Poor	Good	Good
4c Dose skipping	Poor	Good	Good
5. Impact of number of doses in schedule	Poor	Good	Good

### Statistical properties

We expect a good statistical procedure to estimate a specific measure of toxicity and that the precision of this estimate can be gauged. Moreover, we want additional information (i.e., additional patients) to increase the precision in the estimate. Both the model-based and curve-free approach aim to estimate a clear cut measure of toxicity: the dose at which a certain proportion of patients is expected to experience a toxic event. Both methods also quantify the precision of the estimated dose and this precision increases as the sample size increases: they have good statistical properties. Furthermore, were complete information to be available on toxicity of every dose for each patient, the CRM model-based design has performance very close to that of the best design that could exist theoretically [28].

The algorithmic approach, in contrast, tries to find the MTD which is difficult to quantify. As a consequence of this loose definition, it is also impossible to obtain a measure of precision for the MTD. Furthermore, the algorithmic nature of the method means that additional data do not feature in the estimated MTD and the decision about whether a dose is the MTD only depends on what has been observed at this dose level: no learning from the doses above or below is possible. Consequently, algorithmic approaches have very poor statistical properties: the 3 + 3 design correctly identifies the dose with toxicity rate closest to the target level less frequently than model-based designs [29] and over the course of the trial exposes many additional patients to doses above or below that optimal dose. Only 35 % of patients are treated at the optimal dose with the 3 + 3 design compared to 55 % for Bayesian adaptive designs [19].

We gain insight into this poor performance of the algorithmic design by quantifying the evidence when one out of a group of three patients has experienced a DLT: the 95 % confidence interval (CI) around the best estimate of 33 % for the toxicity rate ranges from 0.8 to 90.6 %, illustrating that little has been learned about the true rate. There is not substantially more evidence even when two of six patients studied at a dose experience a DLT: the corresponding 95 % CI for the toxicity rate is (4.3, 77.7 %).

### Simplicity

The ideal method would be easy to describe to clinicians and be straightforward statistically. The latter criterion does not apply to the algorithmic approach as it is not a statistically derived method. It is, however, the simplest to explain to non-statisticians and may be used without the involvement of a statistician. The Bayesian model-based approach is of moderate statistical complexity, but is easily implemented in practice, in part due to software being readily available (e.g., the R package CRM [30]). A particular challenge that does remain is specifying the prior distributions, which we believe is best done in consultation with a statistician. Finally, the curve-free Bayesian method is relatively simple to explain to non-statisticians (though much more complex than the 3 + 3 design) as it directly estimates the risk of each dose rather than using a specific model. The price for this conceptual simplicity is a much more involved statistical process.

The contrast between the model-based and the curve-free approaches is that the former requires the dose–toxicity model to be specified in advance of the study through discussion between the clinicians and a statistician. Nevertheless, it can still consistently identify the dose that has the desired toxicity rate, even when the model has been misspecified [4, 31]. The curve-free approach only assumes non-decreasing risk of toxicity as dose increases. Although this assumption is often reasonable, the curve-free approach depends on it while a model-based approach could allow for decreases in toxicity risk provided that this was specified in the model.

### Intuitive dose recommendations

The optimal dose-escalation method would recommend doses for subsequent patients or for Phase II trials with which an experienced investigator would be comfortable. In general, all three approaches perform well although it does depend on the exact implementation: the originally proposed CRM [4] does sometimes give a counterintuitive dose recommendation [23] but subsequent modifications have been developed [24] to overcome this.

### Flexibility

In particular, we are interested whether any target toxicity rate can be used, how robust the method is to variations in the true dose–toxicity relationship and whether the method can skip doses. The algorithmic approach fairs poorly on all three counts. In its design, the MTD rather than a certain toxicity rate is sought, while dose skipping is prohibited. The dose recommendation only depends on the current dose and hence the underlying dose–toxicity relationship is not exploited. The model-based approach on the other hand caters for any toxicity level and, depending on the exact implementation, can allow dose skipping. In addition, any model can be used although it is clear that specifying the correct model before starting the study will sometimes be difficult. As an alternative to modeling the dose–toxicity relationship, the model-based approach may also be used in a continual reassessment of pharmacokinetic data [13, 18], pharmacodynamic data [32, 33] or toxicity and efficacy data in combination [34]. The Bayesian curve-free approach may also be applied to combined toxicity and efficacy data [35] (and indeed pharmacodynamic data) if these are binary. As well as allowing any target toxicity rate and dose skipping, the biggest advantage of the curve-free approach is that it is suitable for any underlying dose–toxicity relationship provided toxicity increases with dose. This implies that, unlike the model-based approach, the form of the relationship does not have to be specified in advance. Both the model-based and Bayesian curve-free approaches permit single-patient dose cohorts, which can dramatically improve the trial design efficiency.

### Impact of number of doses in schedule

As illustrated in Fig. 1c, a large number of doses allow more precise estimation of the dose of interest although it potentially comes at a price of many patients being treated at sub-optimal doses. With the algorithmic approach, the decision about a dose only depends on what has been observed at this dose level: it does not make use of the information from any of the previous dose levels. In consequence, a large number of doses increase the chance of selecting too safe a dose as even a very safe dose may result in two out of six patients having a DLT [as noted above, the toxicity rate confidence interval in that scenario would be (4.3, 77.7)]. The situation is exacerbated by disallowing skipping of doses and thereby enforcing assessment of every dose incrementally. In contrast, both the model-based and curve-free methods allow dose skipping and use data from all patients in estimating risk of toxicity at each dose level, and so perform well when many doses are available.

## Conclusion

Overall the main benefit of the algorithmic approach is the simplicity of communicating the method and incorporating it into trial protocols and it may be for this reason alone that it is still employed in the vast majority of Phase I trial designs. On the grounds of statistical and other practical considerations, however, it cannot be recommended. The model-based and curve-free approaches have similar merits and are preferable to algorithmic methods due to their superior ability to identify the dose with the desired toxicity rate. Although it is desirable to involve a statistician in the planning of a Phase I oncology trial, Bayesian model-based methods can readily be implemented by a numerate scientist or clinician. The decision whether a model-based approach is to be used will largely depend on the previous information available about the compound under investigation. Substantial evidence from preclinical studies or studies in different indications regarding the shape of the dose–response curve would motivate use of the model-based approach over the curve-free method. If there is sufficient evidence of high enough quality from previous studies, the model-based approach will be slightly superior to the curve-free one, but if not, resulting in the wrong model, it will perform less well.
